# A suspected case of Lyme disease causing complete heart block

**DOI:** 10.1093/omcr/omad089

**Published:** 2023-09-25

**Authors:** Shahin Isha, Rohan Sharma, Rachel M Hannon, Devang K Sanghavi

**Affiliations:** Department of Critical Care Medicine, Mayo Clinic, Jacksonville, Florida, USA; Department of Neurology and Neuro Critical Care, Mayo Clinic, Jacksonville, Florida, USA; Department of Critical Care Medicine, Mayo Clinic, Jacksonville, Florida, USA; Department of Critical Care Medicine, Mayo Clinic, Jacksonville, Florida, USA

## Abstract

Carditis is a rare complication of Lyme disease and usually presents in the early dissemination phase, several weeks after exposure to a tick bite. Conduction abnormalities are the most common manifestation of Lyme carditis. The presentation can vary from atrioventricular conduction delay (first-degree atrioventricular conduction block) to life-threatening situations, such as a complete heart block. Although such manifestations occur late, our case report describes an interesting case where the patient developed a complete heart block in the setting of acute Lyme disease. An elevated IgM with negative IgG Lyme serology titer, an unusual finding, initially led us to face a diagnostic dilemma. Our suspicion of a tick bite was affirmed when we detected a positive titer for *Babesia microti*, an organism carried by the same tick and a common co-infection with Lyme disease. The patient improved with antibiotic therapy and temporary pacemaker support during the initial few days of admission.

## INTRODUCTION

Lyme disease is a tick-borne multisystem disease caused by *Borrelia burgdorferi* (other causative organisms being *Borrelia mayonii*, *Borellia afzelii* and *Borellia garinii*). It is endemically prevalent in the northeastern parts of the USA and some parts of Europe, Japan and China [1, 2]. Clinical manifestations of Lyme disease can be categorized into three phases—early localized (usually within 7–14 d), early disseminated (within days to several weeks) and late disease (several weeks to months) [3, 4]. Lyme carditis usually manifests during the early disseminated phase, occurring within a few weeks from the exposure. Despite being a relatively less frequent complication of Lyme disease, carditis can present with life-threatening presentations, such as bradycardia, heart block, ventricular fibrillation, asystole, etc. [2, 5]. It is thought to be caused by cross-reactive antibodies (IgM and IgG) damaging the heart; however, the exact mechanism is not entirely understood [2]. The most common presentation of Lyme carditis is atrioventricular conduction block (AV block), which can vary from prolonged PR interval to complete AV dissociation [6]. Lyme disease can be diagnosed by detecting Borrelia-specific IgM and IgG antibodies [7]. Our case describes a unique presentation of Lyme carditis associated with a positive IgM but a negative IgG—an uncommon finding.

## CASE REPORT

A 21-year-old man was brought to the emergency department after being found unresponsive and severely bradycardic, with a heart rate in the 40s per minute. He received multiple doses of atropine and IV fluid bolus while being transported to the hospital. On arrival at the Emergency department (ED), he was alert, awake and oriented, with a heart rate of 55/min, blood pressure of 120/70 mm Hg and oxygen saturation of 100%. Initial history revealed no prior medical condition, chest pain, shortness of breath or recreational drug use.

**Figure 1:**
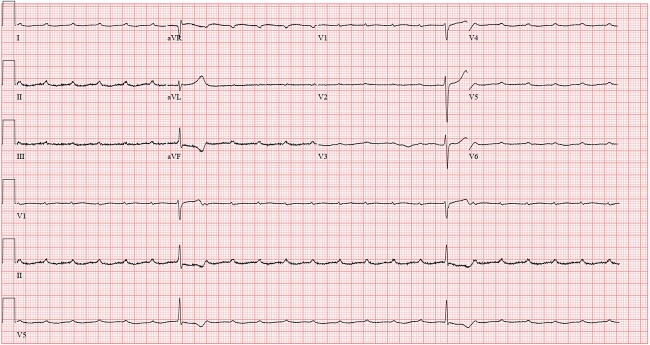
Complete heart block with A-V dissociation (during the initial presentation).

A 12-lead electrocardiogram (ECG) revealed a complete heart block with diffuse T-wave abnormalities (Fig. 1). Serum troponin T at baseline and 2 hours later was normal; NT-Pro BNP was elevated (1623 pg/mL). A complete hemogram showed a hemoglobin level of 14.2 g/dl, a leukocyte count of 8.9 × 10^3^/mm^3^ (Neutrophil 75%, Lymphocyte 13.4%, Monocytes 9%, basophil 0.4%, Eosinophil 0%) and platelet count of 198 × 10^3^/mm^3^. Serum electrolytes and basic metabolic profile were also within the normal range (Na^ +^ 147 mEq/L, K^+^ 3.8 mEq/L, Ca^2+^ 8.7 mg/dl, Mg^2+^ 1.9 mg/dl, PO4^3−^ 3.8 mg/dl, Blood Urea Nitrogen (BUN) 17 mg/dl, Creatinine 1.07 mg/dl) except for an elevated C- reactive protein (CRP) of 17.7 mg/dl. Urine toxicology was unrevealing of any substance abuse. TSH was within normal ranges (3.9 mIU/L). Echocardiography findings were normal, with an ejection fraction of 58%.

While undergoing further evaluation in the ED, he became severely bradycardic with a heart rate in the 20s per min and unresponsive to atropine. Isoproterenol was initiated, which improved the heart rate and associated symptoms. Following a cardiology consultation, he was placed on transcutaneous pacing and later switched to transvenous pacing for complete heart block with atrioventricular (A-V) dissociation. Further exploration of history revealed a visit to a Lyme endemic area 2 weeks before the presentation.

**Figure 2:**
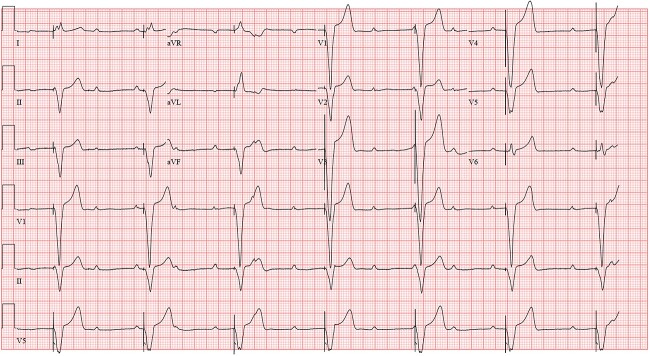
Paced rhythm while on transvenous pacer (on Day 4 of admission).

**Figure 3:**
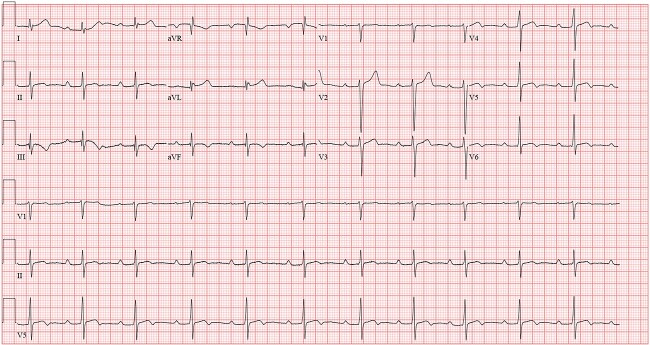
Sinus rhythm with first-degree A-V block (Day 8 of admission).

**Figure 4:**
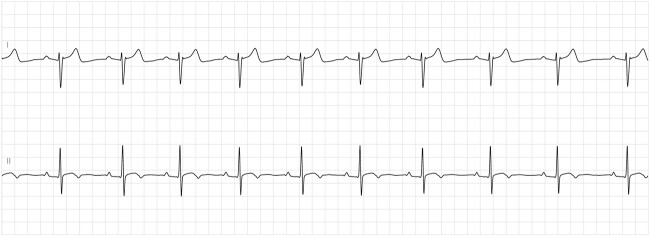
Holter EKG showing sinus rhythm on the day of discharge (Day 9 of admission).

Enzyme immunoassay for Lyme total antibodies was found positive, following which Lyme IgM and IgG were ordered. He tested positive for Lyme IgM antibody, but IgG was negative. After infectious diseases consultation, additional tests for tick-borne infections were done, revealing a positive *Babesia microti* IgG titer. Intravenous Ceftriaxone 2gm daily was initiated. He remained hemodynamically stable in the ICU on transvenous pacing (ECG shown in Fig. 2). The atrioventricular block gradually improved over the next 7 days to the extent that he had 1:1 conduction without needing external pacing (Fig. 3). Considering the satisfactory improvement in his condition, he was discharged with a plan to continue antibiotics until 14 days after the onset of symptoms. A 24-hour Holter report done on the day of discharge showed normal sinus rhythm (Fig. 4) with occasional premature ventricular, and supraventricular complexes, during which he was asymptomatic. He tolerated antibiotic therapy well and did not have any major complaints on his follow-up visit at the infectious disease outpatient clinic.

## DISCUSSION

A sudden onset of complete heart block in a young adult with no prior medical condition raises the suspicion of multiple possible etiologies. In the background of an elevated CRP, high NT-Pro BNP and increased Neutrophil: Lymphocyte ratio, the possibility of myocarditis due to an infectious cause was considered. This patient’s history of a trip to a Lyme endemic region 2 weeks before the presentation led us to pursue the diagnosis of Lyme carditis. His Lyme IgM was positive, but the Lyme IgG was negative, which is a rarely reported finding of Lyme carditis.

The case depicts the importance of a thorough investigation to look for clinical and laboratory-based clues to diagnose an unusual Lyme disease presentation. Although this patient did not have a typical history of rash (erythema migrans), fever or headache before the presentation, we could not entirely exclude the possibility of Lyme disease, considering his trip to a Lyme endemic area. Moreover, the Lyme IgM-positive status is also atypical, as IgM antibodies are produced during the initial few weeks, and carditis usually develops several weeks later after exposure. Despite the diagnostic dilemma, empirical management with parenteral antibiotics was continued, and he showed improvement within the next several days. Another important aspect of his diagnostic workup was the positive *Babesia microtii* titer. Babesia, Anaplasma and Ehrlichia are other organisms that can be spread by the same tick (Ixoded tick) that spreads Lyme disease.

Lyme carditis can occur in 1–10% of Lyme disease cases, usually during the second phase of the disease (early disseminated phase) [1, 2]. Patients may present with typical signs (erythema migrans) and symptoms, but a characteristic history of a tick bite may not be present in many cases [6]. Although myocarditis, endocarditis or pericarditis have been reported infrequently, conduction abnormalities are the most common manifestation of Lyme carditis [2, 5, 8]. Despite a chance of resolving without treatment, it is ideally treated with antibiotics, such as Beta-Lactam (Ceftriaxone) or Tetracycline (Doxycycline) [9]. Patients with severe manifestations, such as hemodynamic instability and high-grade second or third-degree heart block, may need a temporary or permanent pacing device. Lyme carditis generally has a good prognosis, with a resolution of heart block within 7–10 days [10].

## Data Availability

Data sharing is not applicable to this article as no datasets were generated or analyzed during the current study.
